# Pediatric Lymphoma in Jordan: A Retrospective Single-Center Study of Clinical Presentation, Treatment, and Outcomes

**DOI:** 10.7759/cureus.107508

**Published:** 2026-04-21

**Authors:** Mousa A Qatawneh, Moath Altarawneh, Ayman Alhwayan, Bayan Alzghoul, Atwa Altawarh, Suliman Aljaafreh, Haneen Alrawashdeh, Abeer Abdalnabi, Amneh AbuAli, Maher Khader

**Affiliations:** 1 Pediatric Hematology-Oncology, Royal Medical Services, Queen Rania Children’s Hospital, Amman, JOR; 2 Pathology and Laboratory Medicine, Royal Medical Services, Princess Iman Center for Research and Laboratory, Amman, JOR

**Keywords:** biomarkers, child, hodgkin lymphoma, jordan, non hodgkin's lymphoma

## Abstract

Background: Pediatric lymphoma, encompassing Hodgkin lymphoma (HL) and non-Hodgkin lymphoma (NHL), represents a significant cause of childhood cancer morbidity with limited data available from Middle Eastern settings. This study aims to characterize the clinical presentations, biomarker profiles, treatment patterns, and outcomes of pediatric HL and NHL at a tertiary care center in Jordan.

Methods: This retrospective study included children aged 0-16 years diagnosed with HL or NHL at Queen Rania Children’s Hospital in Jordan from 2015 to 2024. Data were collected on demographics, clinical presentation, laboratory markers, treatment regimens, and outcomes. Categorical data were analyzed using chi-square or Fisher’s exact tests, continuous variables using Mann-Whitney U tests, and Event-Free Survival (EFS) was assessed using Kaplan-Meier curves.

Results: A total of 49 patients' data were analyzed. The findings indicate a high rate of late-stage diagnosis among pediatric lymphoma patients in Jordan, with 38 (77.5%) presenting at advanced stages. Most NHL cases involved aggressive subtypes, including Burkitt and T-lymphoblastic lymphoma. Compared with HL patients, those with NHL exhibited significantly higher median C-reactive protein (CRP; 78.5 mg/L vs. 12.0 mg/L, *P* = 0.027) and lactate dehydrogenase (LDH; 553 U/L vs. 355 U/L, *P* = 0.012), indicating greater systemic inflammation and tumor burden in NHL. Among 40 (81.6%) treatment-evaluable patients, chemotherapy achieved a 20 (50%) complete remission rate. Mortality occurred exclusively among NHL patients 13 (26.5%), primarily those with advanced or extranodal disease. Relapse was observed in six (15%) of patients, with a median time to relapse of 37.5 months.

Conclusions: Pediatric lymphoma in Jordan is most often diagnosed at an advanced stage, particularly NHL. Advanced NHL is associated with increased mortality. Early diagnosis, targeted therapy implementation, and incorporation of biomarkers such as LDH and CRP are urgently needed to improve risk assessment and patient outcomes.

## Introduction

Lymphoma accounts for 10% to 15% of all pediatric cancers worldwide [1]. The main groups are Hodgkin lymphoma (HL) and non-Hodgkin lymphoma (NHL). Reed-Sternberg cells, large abnormal lymphocytes, characterize HL [1]. HL includes subtypes such as nodular sclerosis, mixed cellularity, lymphocyte-rich, lymphocyte-depleted, and nodular lymphocyte-predominant HL (NLPHL) [2].

NHL comprises a heterogeneous group of malignancies, several of which are highly aggressive. Key subtypes include Burkitt lymphoma (BL), a rapidly proliferating B-cell tumor; lymphoblastic lymphoma (LBL); diffuse large B-cell lymphoma (DLBCL); and anaplastic large cell lymphoma (ALCL), a T-cell neoplasm characterized by ALK expression [2,3]. These subtypes exhibit distinct clinical features.

Pediatric lymphoma progresses rapidly but often responds well to chemotherapy [4]. In high-income countries, over 90% of patients with HL and 80% to 85% of patients with NHL survive [1,5]. Outcomes are poorer in resource-limited settings. Delayed diagnosis, limited access to treatment, and poor healthcare infrastructure contribute to this discrepancy. This underscores the urgent need for research in these regions to understand how advanced disease at diagnosis and aggressive subtypes impact outcomes.

Inflammatory biomarkers such as C-reactive protein (CRP) and lactate dehydrogenase (LDH) are established prognostic indicators in pediatric malignancies [6]. Elevated CRP levels predict advanced disease and poorer outcomes in children with classical HL [7]. LDH reflects tumor burden across lymphoma subtypes. These accessible markers hold promise for risk stratification in resource-limited settings, yet remain underexplored in Middle Eastern pediatric cohorts.

Pediatric oncology in Jordan has advanced, but research on pediatric lymphoma remains limited, with most studies focusing on histopathological data or general registries that provide little insight into clinical management or outcomes [8,9]. This scarcity hinders the development of tailored care strategies for Jordanian patients. In low-middle-income countries (LMICs), pediatric lymphoma survival lags due to late diagnosis [10]. Our study fills this regional gap by describing clinical presentations, biomarkers, treatments, and outcomes at a major Jordanian center. We aimed to characterize pediatric lymphoma in Jordan with particular attention to disease stage, inflammatory biomarkers, treatment response, and survival outcomes.

## Materials and methods

Study design

We performed a retrospective cohort analysis at Queen Rania Children’s Hospital, the national referral center for pediatric oncology. We reviewed records of pediatric patients aged 0-16 years diagnosed with HL or NHL from January 2015 to December 2024.

Patient selection

Patients included in the study had a confirmed histopathologic diagnosis of lymphoma (HL or NHL). We used computed tomography (CT) and positron emission tomography (PET) scans for initial staging. Our team provided all treatment and regular follow-up with periodic imaging at our institution. We excluded patients whose families declined chemotherapy or chose treatment elsewhere. We also excluded those referred only for bone marrow transplantation and those with incomplete records or follow-up data. Queen Rania Children’s Hospital, the sole center treating military-insured pediatric cancer patients nationwide serves as Jordan's primary public-sector facility for HL/NHL, minimizing selection bias within this cohort.

Data collection

We collected clinical data retrospectively from electronic records and oncology registries. Patient data were anonymized to protect privacy and meet ethical standards. Our data included age at diagnosis, gender, clinical presentation (B symptoms and extranodal involvement), and diagnostic information. We used the Ann Arbor staging system for HL and the St. Jude/Murphy system for NHL. We obtained laboratory results at diagnosis, including LDH, albumin, CRP, erythrocyte sedimentation rate (ESR), hemoglobin, white blood cell (WBC) count, and platelets. Biomarkers (LDH and CRP) were measured at diagnosis using automated immunoassays before treatment initiation. We recorded treatment details, such as chemotherapy, radiotherapy, and bone marrow transplantation. We assessed clinical outcomes, including remission status, relapse, progression, overall survival (OS), and event-free survival (EFS).

Treatment section

Standard institutional protocols were used per international guidelines: COPAD (cyclophosphamide, vincristine, prednisone, doxorubicin) for Burkitt NHL; ABVD (doxorubicin, bleomycin, vinblastine, dacarbazine) for HL; ALL protocol for T-LBL; BEACOPP (bleomycin, etoposide, doxorubicin, cyclophosphamide, vincristine, procarbazine, prednisone), CHOP (cyclophosphamide, doxorubicin, vincristine, prednisone), and ALCL protocols for respective subtypes. Cycle and dose adjustments were made according to local availability and institutional standards.

Outcome measures

OS was defined as the time from diagnosis to death from any cause or to last follow-up. EFS included all patients from the time of diagnosis to the first event (relapse, progression, or death), including pre-treatment mortality, with patients alive without events censored at last follow-up. We assessed treatment response using clinical evaluations, lab testing, and follow-up imaging. Responses were categorized as complete remission (CR), partial remission (PR), stable disease (SD), or progressive disease (PD). Response assessment followed WHO criteria based on clinical, laboratory, and imaging (CT/PET) evaluations at the end of induction. Follow-up consisted of clinical evaluation, laboratory testing, and imaging (CT/PET) every three months during year 1, every six months during years 2 to 3, and annually thereafter, or as clinically indicated. Administrative censoring date: December 2024. Kaplan-Meier curves accounted for right censoring per standard methodology.

Statistical analysis

We analyzed data using IBM SPSS Statistics, version 28 (IBM Corp., Armonk, NY). Categorical variables were expressed as frequencies and percentages. We summarized continuous variables as means with standard deviations (SDs) or medians with ranges/interquartile ranges (IQRs), depending on their distribution. Categorical variables were compared using the chi-square test or Fisher’s exact test. We examined continuous variables using the Mann-Whitney U test. We used Kaplan-Meier curves to analyze EFS and compared HL and NHL groups with the log-rank (Mantel-Cox) test. We considered *P* < 0.05 statistically significant. We did not use Cox regression because all deaths occurred in patients with NHL, making group comparisons unfeasible.

## Results

Forty-nine pediatric lymphoma cases were included: 22 HL (44.9%) and 27 NHL (55.1%). Baseline clinical and laboratory characteristics are summarized in Table 1. No significant differences were found in age at diagnosis (median, 9 years overall; *P* = 0.196) or symptom duration (*P* = 0.760). However, NHL patients had significantly higher CRP (78.5 vs. 12.0 mg/L; *P* = 0.027) and LDH (553 vs. 355 U/L; *P* = 0.012) levels than HL patients; other laboratory parameters showed no significant differences.

**Table 1:** Baseline clinical and laboratory characteristics of pediatric patients with Hodgkin and non-Hodgkin lymphoma. Statistical tests: Mann-Whitney U test for all continuous variables (non-normally distributed data). IQR, interquartile range

Dependent variables	Overall median	Hodgkin lymphoma (*n* = 22, 44.9%), median (Q1-Q3)	Non-Hodgkin lymphoma (*n* = 27, 55.1%), median (Q1-Q3)	Test value	*P*-value
Age at diagnosis/years	9.0	11.0 (7-13)	8.0 (6-11)	-1.293	0.196
Duration of symptoms before diagnosis/weeks	5.0	5.0 (3-9)	4.0 (4-8)	-0.305	0.760
C-reactive protein (CRP, mg/L)	12.0	8 (3-37)	31.5 (8.75-87.25)	-2.215	0.027
Erythrocyte sedimentation rate (ESR, mm/hour)	68.0	67.5 (19.25-82.25)	68.0 (30-95)	-0.674	0.501
Hemoglobin (Hb, g/dL)	11.5	11.25 (9.67-12.37)	11.9 (10.20-13.2)	-1.278	0.201
White blood cells (WBCs, ×10⁹/L)	8.6	9.50 (5.97-16.10)	7.40 (5.7-11.9)	-0.915	0.360
Platelets (PLT, ×10⁹/L)	370.0	364.68 (296.75-435)	400.0 (285-476)	-0.643	0.520
Lactate dehydrogenase (LDH, U/L)	454.0	295.0 (217.25-572.25)	553.0 (328-690)	-2.513	0.012
Albumin (g/L)	38.0	40.0 (31.75-44)	36.0 (26-43)	-0.998	0.318

Demographic and clinical presentation data are summarized in Table 2. Most patients were male (33, 67.3%; *P* = 0.910) and had advanced disease (38, 77.5% stage III/IV; *P* = 0.126). B symptoms occurred in 15 patients (30.6%; *P* = 0.158). Bone marrow involvement was common (38, 77.6%), central nervous system (CNS) involvement was rare (3, 6.1%, NHL only), and extranodal disease was frequent (24, 49.0%; *P* = 0.897), with no significant group differences.

**Table 2:** Demographic and clinical presentation of pediatric patients with Hodgkin and non-Hodgkin lymphoma. *Chi-square test; Fisher’s exact test was used for bone marrow involvement and stage at diagnosis due to expected cell counts <5. CNS, central nervous system; IQR, interquartile range

Dependent variables	Categories	Overall	Hodgkin lymphoma: 22 (44.9%), *n* (%)	Non-Hodgkin lymphoma: 27 (55.1%), *n* (%)	Test value	*P*-value
Gender	Male	33 (67.3%)	15 (68.2)	18 (66.7)	0.013	0.910
	Female	16 (32.7%)	7 (31.8)	9 (33.3)		
B symptoms	Yes	15 (30.6%)	9 (40.9)	6 (22.2)	1.993	0.158
	No	34 (69.4%)	13 (59.1)	21 (77.8)		
Stage at diagnosis	Stage 1	1 (2.0%)	0 (0.0)	1 (3.7)	*χ*² = 5.719*	0.126
	Stage 2	10 (20.4%)	4 (18.2)	6 (22.2)		
	Stage 3	25 (51.0%)	15 (68.2)	10 (37.0)		
	Stage 4	13 (26.5%)	3 (13.6)	10 (37.0)		
Bone marrow involvement	No	38 (77.6%)	18 (81.8)	20 (74.1)	Fisher's exact	0.732
	Yes	11 (22.4%)	4 (18.2)	7 (7.0)		
CNS involvement	No	46 (93.9%)	22 (100.0)	24 (88.9)	-	0.242
	Yes	3 (6.1%)	0 (0.0%)	3 (11.1)		
Lymphadenopathy	No	8 (16.3%)	2 (9.1)	6 (22.2)	-	0.269
	Yes	41 (83.7%)	20 (90.9)	21 (77.8)		
Number of lymph nodes involved	Single	18 (43.9%)	8 (40.0)	10 (47.6)	0.241	0.623
	Multiple	23 (56.1%)	12 (60.0)	11 (52.4)		
Mediastinal	No	38 (77.6%)	17 (77.3)	21 (77.8)	0.002	0.966
	Yes	11 (22.4%)	5 (22.7)	6 (22.2)		
Extranodal involvement	No	25 (51.0%)	11 (50)	14 (51.9)	0.017	0.897
	Yes	24 (49.0%)	11 (50)	13 (48.1)		

**Figure 1:**
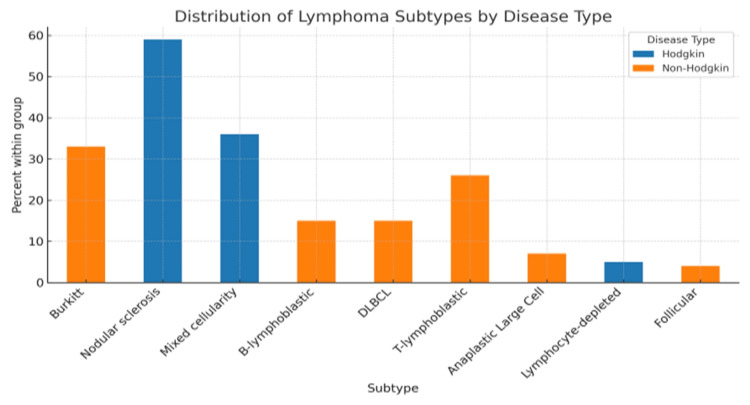
Distribution of lymphoma subtypes among pediatric patients with Hodgkin and non-Hodgkin lymphoma. Values represent the percentage of each subtype within the Hodgkin group (blue bars) or the non-Hodgkin group (orange bars). DLBCL, diffuse large B-cell lymphoma

Histologically, HL cases were predominantly nodular sclerosis (13/22, 59%), followed by mixed cellularity (9/22, 41%). Among patients with NHL, Burkitt lymphoma was most common (10/27, 37%), followed by T-lymphoblastic lymphoma (7/27, 26%). This distribution reflects the predominance of nodular sclerosis in HL and the diversity of Burkitt and T-cell subtypes in NHL (Figure 1).

Treatment modalities and chemotherapy protocols used among pediatric lymphoma patients are summarized in Table 3. Most patients were treated with chemotherapy alone, while radiotherapy was administered to 5 (10.2%) patients and bone marrow transplantation to 3 (6.1%) patients. The most commonly used chemotherapy protocols were COPAD (16, 32.7%), ABVD (15, 30.6%), and the acute lymphoblastic leukemia (ALL) protocol (12, 24.5%).

**Table 3:** Treatment modalities and chemotherapy protocols used among pediatric lymphoma patients. ABVD, doxorubicin, bleomycin, vinblastine, dacarbazine; COPAD, cyclophosphamide, vincristine, prednisone, doxorubicin; ALL, acute lymphoblastic leukemia; BEACOPP, bleomycin, etoposide, doxorubicin, cyclophosphamide, vincristine, procarbazine, prednisone; CHOP, cyclophosphamide, doxorubicin, vincristine, prednisone

Variables	Category	Frequency, *n*	Percentage (%)
Radiation therapy	No	44	89.8
	Yes	5	10.2
Bone marrow transplantation	No	46	93.9
	Yes	3	6.1
Chemotherapy protocol used	ABVD	15	30.6
	COPAD	16	32.7
	ALL protocol	12	24.5
	BEACOPP	2	4.1
	Anaplastic large cell lymphoma protocol	2	4.1
	CHOP	1	2.0
	Surgery then observation	1	2.0

Treatment response and short-term outcomes are summarized in Table 4. Among 40 evaluable patients (81.6%), 20 (50%) achieved complete remission. Relapse occurred in 6 (15%) patients, with a median time to relapse of 37.5 months (range 5-92). Of the 23 first events, 9 (39.1%) were progression, 9 (39.1%) were deaths, and 5 (21.7%) were relapses. Median EFS was four months (range 1-92). Among 49 patients, 9 (18.4%) died before treatment response assessment.

**Table 4:** Treatment response and outcomes of pediatric lymphoma patients. Among 49 patients, 9 (18.4%) died before treatment response assessment.

Variable	Category	n	Percentage (%)
Response to initial therapy (*N* = 40)	Complete remission	20	50.0
	Partial remission	9	22.5
	Progressive disease	7	17.5
	Stable disease	4	10.0
Diagnosis to first event (*N* = 23)	Relapse	5	21.7
	Progression	9	39.1
	Death	9	39.1
Relapse	No	34	85.0
	Yes	6	15.0
Mortality	No	36	73.5
	Yes	13	26.5

EFS did not differ significantly between the HL (mean 39.7 months) and NHL (mean 60.5 months) groups (log-rank *P* = 0.885), indicating similar outcomes. The median EFS was four months (range 1-92 months) for the 23 (47%) patients experiencing first events (9 (39%) pre-treatment deaths, 9 (39%) progression, and 5 (22%) relapse); 26 (53%) patients were censored event-free at last follow-up. All 13 (26.5%) deaths occurred in NHL patients with advanced disease (8/13, 61.5%) and frequent extranodal involvement (10/13, 76.9%). Patient characteristics among the deceased are summarized in Table 5. Among the deceased, the mean age at diagnosis was 9.4 years (SD 3.9; range 2-16), and the median overall survival after diagnosis was nine months (range 1-104).

**Table 5:** Characteristics of pediatric patients with lymphoma who died during follow-up (N = 13). DLBCL, diffuse large B-cell lymphoma

Variables	Category	*n* (*N* =13)	Percentage (%)
Gender	Male	9	69.2
	Female	4	30.8
Lymphoma subtypes	Hodgkin	0	0
	Non-Hodgkin	13	100
Lymphoma subtypes	Burkitt	5	38.5
	DLBCL	4	30.8
	T-lymphoblastic lymphoma	4	30.8
Stage	Stage 3	5	38.5
	Stage 4	8	61.5
B symptoms	Yes	4	30.8
	No	9	69.2
Extranodal involvement	Yes	10	76.9
	No	3	23.1

## Discussion

This study specifically addresses pediatric lymphoma in Jordan, focusing on its clinical features and treatment outcomes in this population. NHL and HL had similar prevalence. One of the most interesting aspects of this study was the relatively high proportion of patients with advanced-stage disease at the time of diagnosis: 25 (51%) were diagnosed at stage III and 13 (26.5%) at stage IV. These results highlight the issue of early detection in Jordan, a problem corroborated by other studies [11].

The treatment strategy developed in this study primarily focused on chemotherapy. It was notable that 20/40 (50%) of patients with aggressive disease achieved complete remission. However, the most alarming finding was the high mortality rate of 13 (26.5%), which was observed only in the NHL group, particularly among patients with advanced-stage disease and extranodal involvement. The short median EFS (4 months) reflects early treatment failure or mortality in advanced NHL (38, 77.5% late-stage), consistent with LMIC challenges. Late relapses (median 37.5 months) occurred only in responders, highlighting divergent risk trajectories within the cohort. These results clearly indicate that aggressive forms of NHL, such as Burkitt and T-lymphoblastic lymphoma, continue to pose a substantial treatment challenge [12].

In this cohort, despite the relatively low rates of radiotherapy 5 (10.2%) and bone marrow transplantation 3 (6.1%), which fall short of the clinical guideline stating that chemotherapy is the first-line therapy, this appears to represent a lack of therapeutic strategy. When the focus is on the long-term complications of treatment, the aim is to decrease them. This consideration is particularly relevant for pediatric patients who may experience treatment-related sequelae into adulthood.

Comparative international studies show the same pattern with advanced-stage diagnosis. In upper middle-income and high-income countries, early diagnosis and treatment provide the highest survival rates, reaching upwards of 90% for HL and 80%-85% for various subtypes of NHL [13]. On the other hand, middle- and low-income countries have been shown to have significantly poorer survival rates, even when offered the same treatment approaches [12]. This study demonstrates high mortality rates among patients with NHL, which illustrates the clear lack of early intervention and treatment for aggressive subtypes, such as Burkitt lymphoma, which have been shown to worsen rapidly without adequate treatment [11].

Even more critical is the need to recognize subtypes of NHL, which are most aggressive and more easily managed at early stages of the disease. Our study identified significantly elevated CRP (median 78.5 vs. 12.0 mg/L, *P* = 0.027) and LDH (553 vs. 355 U/L, *P* = 0.012) levels in NHL compared to patients with HL. These inflammatory biomarkers have established prognostic value in pediatric lymphoma [6,7].

Elevated CRP correlates with advanced stage and B symptoms in childhood HL, while LDH reflects tumor burden and independently predicts inferior event-free survival, particularly when >500 U/L [14]. Notably, all 13 (26.5%) deaths occurred exclusively in the NHL group, characterized by higher biomarker levels, advanced stage (8/13, 61.5% stage IV among fatalities), and aggressive subtypes (Burkitt 5/13, 38.5%; T-LBL 4/13, 30.8%). This pattern aligns with international pediatric trials, in which LDH ≥500 U/L identifies patients with high-risk B-NHL who require treatment intensification [15].

Consideration of these study findings within the context of pediatric lymphoma management in Jordan and other comparable countries demonstrates clear value. The overwhelming rates of diagnoses made at an advanced stage of disease support the need for much stronger efforts to detect the disease at early stages. Improved public education, removal of bureaucratic hold-ups to access care, and advanced professional education on early disease recognition work in the specialist’s favor, even for these challenging patients.

This study has several limitations. As a retrospective study, reliance on medical records can lead to missing or incomplete data. The pediatric lymphoma cases originated from a single institution (Queen Rania Children’s Hospital) and do not represent Jordan’s entire pediatric population, restricting external validity.

The small sample size (*n* = 49) limited power for some comparisons, though post-hoc analysis confirmed adequate power for significant biomarkers (CRP 99.5%, LDH 92.2%; G*Power, α = 0.05). Treatment heterogeneity (COPAD/ABVD across subtypes), all 13 (26.5%) deaths occurring in NHL, and potential selection bias (despite a military-insured catchment minimizing this) precluded adjusted survival analyses. Biomarker differences are exploratory and descriptive. Multicenter studies are needed for validation.

## Conclusions

This single-center retrospective study (*n* = 49) at Queen Rania Children’s Hospital documents advanced-stage presentation (38, 77.5%) and high NHL mortality (13, 26.5%) among pediatric lymphoma patients in Jordan. Despite achieving complete remission in 20/40 (50%) of treatment-evaluable patients, early treatment failures and disease progression remain significant challenges. These findings highlight the need for future multicenter research to investigate early diagnosis strategies, biomarker-guided risk stratification, and optimized treatment protocols for pediatric lymphoma in Jordanian and regional healthcare settings. Larger studies incorporating national registry data would enhance generalizability and inform targeted interventions.
